# Mothers and mosquitoes: climate change contributes to the spread of vector-borne pathogens posing a substantial threat to pregnant women

**DOI:** 10.1007/s00281-025-01050-z

**Published:** 2025-04-24

**Authors:** Pauline Wiemers, Isabel Graf, Marylyn M. Addo, Petra C. Arck, Anke Diemert

**Affiliations:** 1 Division of Experimental Feto-Maternal Medicine, Department of Obstetrics and Fetal Medicine, University Medical Center Hamburg-Eppendorf, Hamburg, Germany; 2 Hamburg Center for Translational Immunology, University Medical Center Hamburg-Eppendorf, Hamburg, Germany; 3 Institute for Infection Research and Vaccine Development, University Medical Centre Hamburg-Eppendorf, Hamburg, Germany; 4 Department for Clinical Immunology of Infectious Diseases, Bernhard Nocht Institute for Tropical Medicine, Hamburg, Germany; 5 German Center for Infection Research, Partner Site Hamburg-Lübeck-Borstel-Riems, Hamburg, Germany; 6 German Center for Child and Adolescent Health, Partner Site Hamburg, Hamburg, Germany; 7 Department of Obstetrics and Fetal Medicine, University Medical Center Hamburg-Eppendorf, Martinistrasse 52, 20251 Hamburg, Germany

**Keywords:** Vertical transmission, Fetal infection, Maternal immune activation, Maternal infection, Guidelines, Climate change, Vector-borne diseases

## Abstract

Infectious diseases have threatened individuals and societies since the dawn of humanity. Certain population groups, including pregnant women, young children and the elderly, are particularly vulnerable to severe infections. Over the past few centuries, advances in medical standards and the availability of vaccines have reduced infection-related mortality and morbidity rates in industrialized countries. However, the global rise in temperatures and increased precipitation present a new challenge, facilitating the broader distribution of disease vectors, such as mosquitoes, bugs and ticks, to higher altitudes and latitudes. Consequently, epidemic and pandemic outbreaks associated with these vectors, such as Zika, West Nile, dengue, yellow fever, chikungunya and malaria, are increasingly impacting diverse populations. This review comprehensively examines how infections associated with climate change disproportionately affect the health and well-being of pregnant women and their unborn children. There has been a noticeable emergence of vector-borne diseases in Europe. Consequently, we stress the importance of implementing measures that effectively protect pregnant women from these increasing infections globally and regionally. We advocate for initiatives to safeguard pregnant women from these emerging threats, beginning with enhanced education to raise awareness about the evolving risks this particularly vulnerable population faces.

## Introduction

Humanity has faced infections for thousands of years, with notable examples including the plague dating back 5,000 years and now considered extinct [1]. Yet, recent and ongoing endemics and pandemics have been recorded over the last centuries, e.g., caused by influenza or Coronavirus [2]. To this day, infectious diseases remain among the leading causes of morbidity and mortality globally, with a particularly severe impact in developing countries [2]. Although infection-related deaths have decreased significantly in recent years due to advances in vaccination, therapeutic options, medical care and hygiene practices, the SARS-CoV-2 pandemic exemplifies the profound effect that emerging pathogens can have on individuals, healthcare systems and societies. COVID- 19 alone has resulted in more than 6 million deaths worldwide [3].

**Fig. 1:**
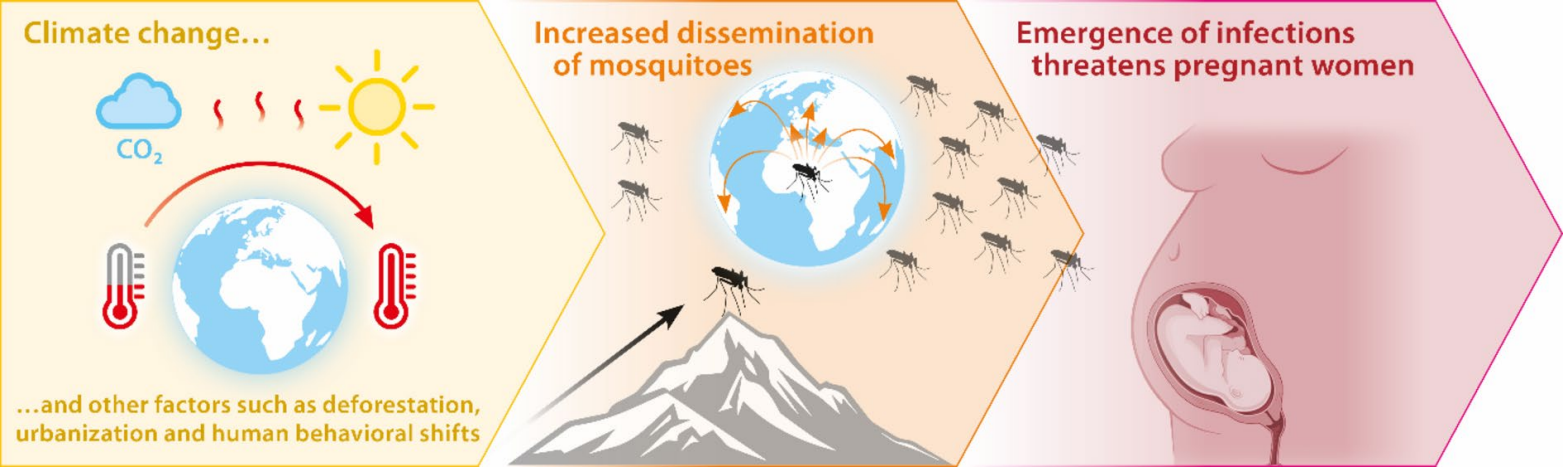
The emergence of vector-borne diseases is mediated by a variety of factors including urbanization, deforestation, human behavioral shifts in migration and consequences of globalization such as increased traveling behavior. Besides, rising global temperatures and changing precipitation patterns associated with climate change are of big relevance as they enhance the replication rates of disease-carrying vectors, such as mosquitoes and other arthropod species. This extends the transmission periods at higher altitudes and latitudes and accelerates the replication rates of pathogens, including viruses and parasites. The resulting infections pose significant health risks, particularly affecting vulnerable populations such as pregnant women and their unborn children.

Humanity is currently contending with another significant factor facilitating the transmission of infectious diseases: the accelerating impacts of climate change [4]. This phenomenon manifests through rising global temperatures, alterations in precipitation patterns and increased natural disasters such as droughts and floods. The escalating temperatures also influence the habitat, behavior and reproductive traits of disease vectors relevant to humans, particularly mosquitoes and other arthropod species. As cold-blooded organisms, mosquitoes are susceptible to temperature increases, which enhances their survival rates, boosts replication efficiency rates, and modifies their biting behavior and virus replication rates [5–7]. The rising temperatures create new habitats and extend transmission periods at higher altitudes and latitudes [8] (Fig. 1). Moreover, climate change disrupts human activities, further elevating the risk of infection. Floods and droughts can affect land and pesticide use, prompting migration to urban areas and increasing population density [5]. The intricate relationship between host density, land use, urbanization, human movements and disease vectors has been explored in great detail [5, 9]. It is well known that animals act as reservoirs of pathogens, e.g., mosquitoes, as discussed here. Furthermore, migrating animals, such as birds, disseminate pathogens. Intriguingly, humans can also act as reservoirs and carriers of infectious pathogens, e.g. in the context of migration and travelling, hereby directly exposing other human populations in target regions to diseases to which their immune systems are not yet adapted [10].

Emerging infections pose significant risks, particularly for specific population groups such as the elderly, children and pregnant women [11]. Due to the physiological adaptations of the immune system during pregnancy, pregnant individuals exhibit a markedly increased susceptibility towards infections [12]. The severe course of infections during pregnancy not only endangers maternal health but also represents a potential threat to the unborn child, thereby compromizing the child’s future health and well-being [13]. While numerous infectious diseases linked to pregnancy have been the subject of extensive scientific research, the risks posed by vector-borne infections to both mother and child—particularly in the context of climate change—remain significantly underexplored [14]. Studies and empirical data regarding the clinical and immunological manifestation of these infections during gestation are scarce. Thus, this review highlights recent findings on the emergence of new vector-borne infections triggered by climate change and their impact on pregnant individuals, emphasizing the most prevalent diseases transmitted by mosquitoes. Many of the mosquito-borne infections we here discuss are endemic in regions such as Africa, Asia, South America, and Oceania. Climatic changes have a significant potential to worsen the disease burden in these endemic regions in the near future by facilitating a greater dissemination, e.g. to higher altitudes [15–17]. This review, however, focuses primarily on the dissemination of vectors into Europe and the related threats for pregnant women caused by the emerging infections.

### Climate change accelerates the transformation of the infectious disease landscape

Pathogens pose significant and evolving threats through two primary mechanisms: the emergence of novel strains within a population, and the enhanced virulence of pre-existing strains. These processes can lead to a marked increase in incidence rates or facilitate an expansion of the geographic distribution of these pathogens, thereby impacting public health on multiple levels [18]. The emergence of new infections is influenced by various factors, including environmental changes such as deforestation and climate change, behavioral shifts in migration, increased travel and globalization [17, 19, 20]. Limited access to healthcare systems, particularly during crises and conflicts, can significantly contribute to the spread of infectious agents [21]. Additionally, the rising issue of drug resistance and the slow development or distribution of vaccines further accelerate the emergence of infections [22]. Moreover, spontaneous or environmentally induced mutations of viruses, for example, can facilitate the more rapid transmission between hosts or even to different species [2].

To date, we have observed that climatic changes have favored the emergence of a diverse array of the arthropod family – including mosquitoes and ticks [8, 23]. This development has, for instance, contributed to a rise in cases of Lyme disease caused by the transmission of spirochetes through ticks, which has become the most prevalent vector-borne disease in Europe [24, 25]. In recent years, there has been a concerning rise in diseases previously endemic to other regions but largely absent in Europe, a phenomenon attributed to the increasing spread of vectors such as mosquitoes. This spread can be attributed to climate change, globalization, and changes in land use, which facilitate the dissemination of these vectors into Europe [8, 26]. The implications for public health are significant, necessitating a reevaluation of European disease prevention and control strategies.

Among arthropods, the Asian tiger mosquito (*Aedes albopticus*) and the yellow fever mosquito (*Aedes aegypti*) are particularly relevant in this context. They transmit pathogens of the arbovirus family, such as dengue, Zika, Chikungunya, West Nile fever and yellow fever viruses. Other mosquitoes of the *Anopheles* species transmit the tropical disease malaria via plasmodia. While this review primarily addresses the more prevalent vector-borne diseases, it is essential to acknowledge that a diverse array of additional pathogens can also be transmitted by mosquitoes, as delineated in Table 1. The potential for an increase in these infectious diseases may be influenced by changing climatic conditions, which should be considered in future research initiatives.

**Table 1:** Mosquito-borne diseases with low prevalence may disseminate and become a potential threat to maternal and newborn health in other regions in the future

Pathogen	Disease caused	Cases in Europe
Oropouche virus	Oropouche fever	19 travel-related cases were registered in 2024 [27]
Rift Valley virus	Rift valley fever	There was one probable case in France 2021 [28]
O’nyong’nyong virus	O’nyong’nyong fever	Sporadic travel-related case reported [29]
Japanese encephalitis virus	Japanese encephalitis	Sporadic case reports [30]
Parasitic roundworms	Lymphatic filariasis	Sporadic migration-related cases [31]
Venezuelan equine encephalitis virus	Venezuelan equine encephalitis	No recent cases reported

### Pregnancy as a risk factor for a severe course of infection

Pregnant women are especially susceptible to infections, a phenomenon that can be partly attributed to the physiological adaptations to pregnancy. These adaptations include an enhanced blood supply to mucosal tissues and the decreased lung volume resulting from the diaphragmatic elevation [32]. Additionally, the maternal immune system undergoes adaptations that foster a tolerogenic environment, which plays a significant role in the increased severity of infections during pregnancy [33, 34]. The net outcome of this immune tolerance mounted during pregnancy is the suppression of anti-fetal effector responses, allowing for the survival of the semi-allogenic fetus. Research on influenza infections during pregnancy—despite not being a vector-borne disease—reveals that the unique temporary immunological state during pregnancy not only avoids rejection of the semiallogenic fetus but also hinders the innate and adaptive immune responses to mount an effective anti-pathogen response. This ineffective elimination of pathogens is considered to be one of the main factors contributing to the severity of infections observed in pregnant women. More specifically, the less efficient anti-viral response mechanisms during pregnancy result from an altered antigen presentation capacity of innate immune cells, lower frequencies and efficiency of the pathogen-specific T cell response, reduced migration of effector cells to the site of infection, such as the lung, and an increased mutation rate of pathogens due to lower environmental pressure in the host [34]. This less efficient anti-viral immune response to virus pathogens causes high maternal morbidity and mortality, along with severe perinatal complications. The latter include an increased risk for pre-eclampsia, stillbirths and miscarriages, intrauterine growth restriction, birth defects, low birth weight and premature birth on the part of the fetus [33, 35, 36].

Moreover, not only the morbidity but also the mortality of pregnant women shows striking differences compared to non-pregnant women. For example, the mortality rate observed during the various IAV pandemics was higher in pregnant women compared to non-pregnant women [35].

Infection with IAV is just one example of a pulmonary infection that can unfavorably affect maternal health and the course of pregnancy. Another example is the infection caused by coronavirus strains, such as severe acute respiratory syndrome coronavirus 2 (SARS-CoV-2). The COVID-19 pandemic has underscored that infection with specific SARS-CoV-2 virus substrains can lead to severe illnesses in pregnant women, accompanied by a higher risk of fetal loss or preterm birth [37, 38]. In addition to these airway infections, other viruses, such as cytomegalovirus (CMV), which can be transmitted through contact with young children, are of significant concern during pregnancy. While the global seroprevalence of CMV in the general population is approximately 83% and maternal infection often occurs asymptomatically, it poses a serious risk due to a high rate of vertical transmission to the fetus [39, 40]. This increases the likelihood of long-lasting neurological disabilities as well as vision and hearing loss in the offspring.

However, for mosquito-transmitted diseases, pregnancy as a severe course of infection is less well studied which highlights the urgency to advance research in this field.

### Emerging vector-borne challenges: the rising incidence of mosquito-borne diseases across the European continent

The rising incidence of infections and the broader propagation of diseases can be attributed to various factors. More specifically, the prevalence of travel- and trade-related infectious cases has consistently increased over the past decade, as evidenced by extensive surveillance data [41]. In principle, the rising infection rates follow a discernible pattern: vectors like mosquitoes, which trigger endemic diseases primarily in tropical and subtropical regions, are increasingly disseminated by travelers and traders due to globalization [19]. Driven by the climate crisis—with rising temperatures and humidity—favorable mosquito survival conditions are emerging in previously unaffected area [17, 41, 42]. Within Europe, countries in southern and central regions are experiencing heightened impact, and it is expected that the dissemination and persistence of mosquitoes will extend to northern Europe [43, 44]. The following sections will focus on mosquito-borne diseases that pose the greatest threat to pregnant individuals.

A critical vector responsible for transmission is the Aedes mosquito, which carries various viruses from the Flaviviridae family, such as Zika virus, dengue virus and yellow fever virus. Zika virus was first isolated in Africa in 1947 and remained confined to the African continent for decades [45]. The Zika pandemic in Brazil between 2015 and 2017, which affected over one million people, led to global recognition of the threat posed by this pathogen [46]. This was the first instance in which the teratogenic effects of Zika virus infection during gestation were brought to the attention of society [47]. At that time, over 3500 cases of congenital Zika syndrome were recorded [48]. In recent years, the European Centre for Disease Prevention and Control (ECDC) has been monitoring and documenting cases of Zika infections across Europe. In this context, infections have been identified in countries such as Germany, France, and Spain [49]. The peak of these infections occurred in 2016, coinciding with the Zika pandemic in Brazil. It is suggested that the Zika cases reported in Europe were travel-related and, consequently, a result of globalization. Similarly, the chikungunya virus - an alphavirus from the Togaviridae family—was also imported by travelers and traders and has since spread to several industrialized countries, causing outbreaks in the Americas and Europe [50, 51]. In 2007, chikungunya virus was first introduced to Europe by travelers, leading to an initial outbreak that predominantly affected Italy [52]. By 2017, chikungunya virus infections were confirmed in ten European countries, impacting Italy and the United Kingdom [53]. Notably, over half of the cases reported in Europe were locally acquired infections rather than travel-related [50], resulting from favourable climate conditions that promote the survival of *Aedes* mosquitoes. As a result, it is assumed that the *Aedes* mosquito has locally established itself in Europe [54].

Yellow fever virus and dengue virus are endemic to several tropical and subtropical regions, including Africa and South America, with approximately 200,000 infections of yellow fever annually and a ten-fold increase in worldwide case numbers of dengue infections registered in 2019 (5.2 million) compared to 2000 [55]. Throughout the era of the slave trade, which continued until the 19 th century, outbreaks of yellow fever were also documented in Europe [56]. Global trade and enhanced travel behaviors have facilitated the spread of one of the vectors for yellow fever virus, *Aedes albopictus*, into various European countries, including the Netherlands, Germany, Switzerland, the UK, and numerous southern to mid-European nations [57]. Notably, yellow fever virus has been detected in the saliva of *Aedes albopticus* mosquitoes in France, indicating the potential transmission capacity [58]. Projections suggest that yellow fever virus will continue to disseminate in Europe in the coming years [59]. A mathematical model assessing the future distribution of *Ae. aegypti* in Europe under various climate change scenarios anticipates the presence of this mosquito species along the Mediterranean coast by 2090 [60].

Another flavivirus infection, the West Nile virus, is also increasingly prevalent. Birds and other animals serve as hosts of the West Nile virus, whilst transmission to humans occurs via *Culicidae* mosquitoes. First identified in Uganda in 1937, West Nile virus caused sporadic outbreaks of mild infection primarily in Africa, the Middle East, Russia and Europe over the subsequent decades [61, 62]. These were predominantly caused by the strain West Nile Virus-2. In the 1990s, a new West Nile Virus-1 strain emerged in countries such as Russia and Israel, leading to severe illnesses characterized by neurological symptoms [42]. Over the past 20 years, this virus strain has spread to the United States and neighboring regions, such as Canada, Mexico, the Caribbean and Southern America. Additionally, it has reached several European countries, particularly in Southern Europe, where cases peaked in 2018. Central European countries, including the Netherlands and Germany, report West Nile virus infection cases yearly [63]. Looking ahead, a broader spread of flaviviruses, such as West Nile virus, is generally anticipated [55].

Besides viruses, parasites can also be transmitted by mosquitoes. A well-known example is malaria, an infection primarily spread by *Anopheles* mosquitoes. Malaria can be caused by five types of parasites, most commonly by *Plasmodium falciparum* and *Plasmodium vivax*. It is predominantly endemic in Africa, where approximately 94% of cases are found [64]. However, malaria also impacts populations in South America and large parts of Asia [65]. The WHO reports that in 2022, 249 million people were infected with malaria worldwide, of which approximately 608,000 died [64]. Children under the age of five are particularly vulnerable to increased morbidity and mortality from malaria, as are immunosuppressed individuals and pregnant women [66]. In the 2023 malaria report, the WHO emphasizes that climate change exacerbates the global malaria burden. Rising temperatures from heat waves, along with increased precipitation leading to humidity and flooding, create more favorable conditions for the survival and spread of *Anopheles* [64]. Even a slight increase in temperature can lead to malaria becoming endemic in regions that are currently malaria-free. This applies not only to horizontal dissemination but also to the vertical expansion of *Anopheles* into higher altitudes [67]. At present, malaria cases in Europe are predominantly travel-related; however, locally acquired infections have also been reported in European countries, such as France, Germany, Spain, and Ireland. Nevertheless, these infections are mostly linked to travel, as airport staff have been affected [68]. It has been suggested that changes in precipitation and rising temperatures due to climate change will enable a northward spread of *Anopheles* mosquitoes [69], potentially making malaria endemic in Europe.

### Maternal and fetal effects of infection during pregnancy

#### Virus-induced infections during pregnancy

The mosquito-borne arboviruses discussed in this review primarily cause asymptomatic or mild disease in non-pregnant individuals. Typical clinical presentations include fever and flu-like symptoms, as well as hemorrhagic fever in the cases of yellow fever and dengue. Yellow fever stands out as it can lead to a severe disease progression, with approximately 15% of infected individuals experiencing febrile temperatures, often accompanied by jaundice due to virus-induced hepatitis and renal failure [70]. Under certain conditions, severe bleeding, life-threatening shock symptoms, and multi-organ failure may also arise, resulting in a high mortality rate of up to 60% among those affected [55, 70, 71].

**Fig. 2:**
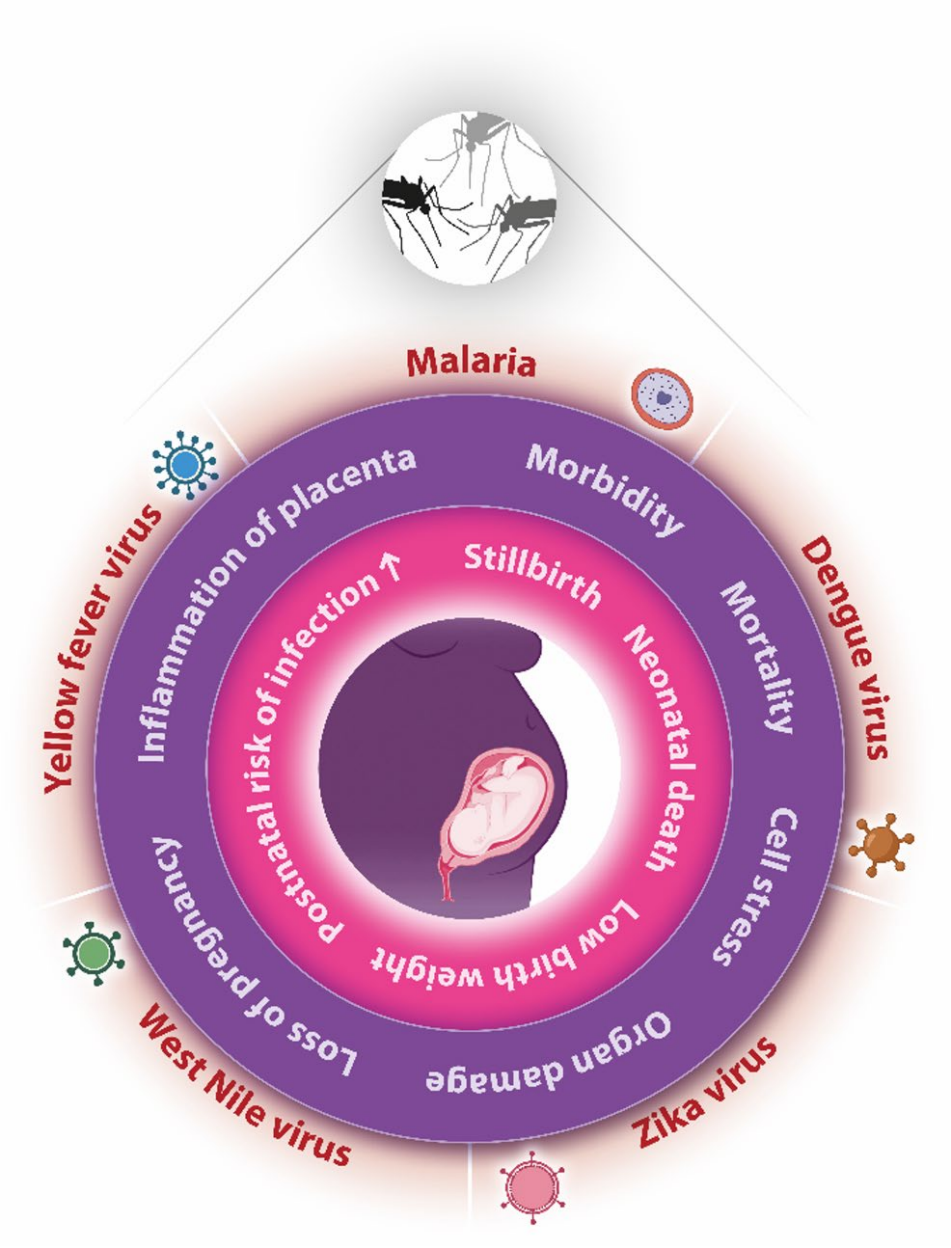
Generalized overview of the potential effects of vector-borne diseases on maternal and fetal health. The growing prevalence of pathogens transmitted by a variety of mosquito species can pose significant health risks for pregnant women and their developing fetuses. Addressing these challenges requires a comprehensive understanding of how climate-related changes influence pathogen dynamics and the implications for reproductive health outcomes. Purple = Implications of vector-borne infections on maternal health. Pink = Implications of vector-borne infections on fetal health

During pregnancy, infection with Zika or dengue increases the risk of maternal and fetal complications, as summarized in Fig. 2, whereas the data on West Nile and yellow fever infections are somewhat ambiguous and sparse. In the case of Zika, the virus can be transmitted to the fetus, as observed in approximately 20–40% of pregnancies [72, 73]. Interestingly, Zika virus can also selectively infect the placenta [74]. Zika infection triggers an inflammatory immune response at the feto-maternal interface, which likely accounts for the observed increase in pregnancy complications [75]. These mainly affect the unborn child and include structural damage to the brain and microcephaly in approximately 20% of fetuses that were infected with Zika via vertical transmission [76]. Here, the time point of infection seems to be essential as teratogenicity of Zika virus is documented when infection occurs in the first or second trimester [77]. In contrast, infections later during pregnancy have other manifestations. The congenital damages to the brain include e.g. calcifications, enlarged ventricles and reduced brain volumes, which can be paralleled by severe neurosensory impairments such as eye lesions, hearing abnormalities and musculoskeletal lesions [78]. These abnormalities have been associated with increased cytokines in placental tissue and amniotic fluid, e.g., interleukin (IL)-6, IL-15, IL-17 [79]. Other studies report that infection with Zika may inhibit autophagy and trigger apoptosis in neuronal progenitor cells, critical pathways during neuronal development. Insights from preclinical models highlight that neonatal Zika virus infection leads to an overshooting CD8+ T cell-mediated proinflammatory response, which could be linked to the degeneration of neuronal cells [80].

Most studies aiming to assess the inflammatory response during Zika infection have focused on the analysis of placental tissue and the reproductive tract. Consequently, information on systemic inflammation in infected pregnant women remains sparse; this knowledge gap should be urgently addressed in future research studies.

Zika infection during pregnancy also increases the risk for fetal loss, which occurs in approx. 10% of all maternal-fetal transmissions [73, 76]. The pathogenesis of this severe pregnancy complication is still unknown. Still, it may be explained by the cytokine surge at the feto-maternal interface, which could lead to the rejection of a potentially viable fetus despite possible congenital brain damage. It is well known that an overshooting inflammatory response can cause pregnancy complications such as preterm birth [81]. One could also hypothesize that the congenital brain damage leads to fetal distress, which may initiate maternal rejection. More work is needed to test these hypotheses, which is urgently required due to the endemic presence of Zika in an increasing number of continents.

Infection with dengue during pregnancy can cause perinatal complications, such as stillbirth and neonatal mortality [82]. Other perinatal complications, such as preterm birth and low birth weight, have been proposed [83, 84]. The lack of clinical studies with a large number of participants and the bias induced by studies with few participants hamper the estimation of the risk for these perinatal complications. In a recent meta-analysis with over 39.500 dengue-infected patients, the authors report no statistically significant difference between dengue-infected and non-infected pregnant women concerning the risk for preterm birth and low birth weight [82]. However, this meta-analysis highlighted that dengue infection during pregnancy increases maternal morbidity and even mortality. Pregnant women are at higher risk of developing the so-called dengue shock syndrome, which affects almost 15% of pregnant women compared to 5% of the non-pregnant population. Thrombocytopenia, cell stress or even liver damage may account for the severe clinical cause of dengue infection in pregnant women [85, 86]. However, the risk for dengue shock syndrome in non-pregnant individuals was not stratified by sex. This is critical since females are at higher risk than males of developing dengue shock syndrome [87]. Hence, the difference in dengue shock syndrome between non-pregnant and pregnant women may be more negligible than reported.

The vertical transmission of dengue from mother to child could be shown within prospective cohort studies [88–90]. However, the transmission rate shows a significant variation between the different studies. Still, infection of the placenta and transmission to the fetus might account for one possible mechanism favoring adverse perinatal outcomes. Apart from that, similar to other infections, dengue virus infection in non-pregnant patients was associated with an inflammatory response, such as higher levels of IL-8, IL-6 and tumor necrosis factor (TNF) [91, 92]. This surge of inflammation may account for dengue fever-related pregnancy complications [12, 81]. Also, the cytokines elevation upon severe dengue infection has increased vascular permeability and plasma leakage [93]. These mechanisms have not yet been investigated in the context of pregnancy but may account for insufficient placental perfusion.

Data on the implications of West Nile fever on pregnancy are somewhat ambiguous and sparse. West Nile virus infection during pregnancy has been reported to cause severe symptoms, such as fever, nausea, meningism and even seizures; the latter indicates inflammation of the CNS [94–96]. Given the effects of other flaviviruses, such as Zika virus, on pregnancy outcomes, potential teratogenic effects and adverse pregnancy outcomes are also vividly discussed in the context of West Nile virus-induced infection in pregnant women. Published data in this regard are still ambiguous, ranging from the report of birth defects in one study to no evidence for adverse birth outcomes in others [55, 94, 97]. In a case report, intrauterine West Nile virus infection – confirmed by West Nile virus-specific IgM in fetal serum and cerebrospinal fluid - resulted in bilateral chorioretinitis and also severe neurological defects, such as a loss of white matter and cystic modifications [98]. In another study, which included more than 70 pregnant women infected with West Nile virus, the potential risk for congenital disabilities could not be confirmed. However, this study is limited because the vertical transmission could not be confirmed [97]. Overall, evidence for vertical West Nile virus transmission is sparse. One study reports a transmission rate of 4%, along with an increased risk for fetal encephalic changes [94]. However, the number of participants in these studies is still too low, and studies often lack information on seroconversion during pregnancy, which limits the ability to draw conclusions.

In contrast to the previous virus diseases, yellow fever infection can already lead to serious consequences in non-pregnant women, making it essential to investigate the potential threat of yellow fever infection during pregnancy. Surprisingly, this has been relatively neglected in studies published to date. Emerging evidence suggests the vertical transmission of yellow fever virus in two cases, confirmed by yellow fever virus-specific IgM antibodies or PCR. Here, the children were initially asymptomatic at birth [55, 99, 100]. Later, they suffered from fever, accompanied by multiorgan failure and coagulopathy and consequently developed a fatal outcome.

Lastly, chikungunya stands out as an infection as it does not seem to enhance the risk of pregnancy complications or adverse fetal outcomes compared to non-infected pregnant women [101, 102]. However, the elderly and newborns are known to be particularly vulnerable to the severe course of chikungunya-induced infections, which can potentially result in fatal outcomes [103, 104]. Intriguingly, pregnant women seem to be protected from joint pain during chikungunya infection [102]. Opposed to the mild course of chikungunya infection in pregnant women, encephalopathy and a high morbidity rate were observed in neonates perinatally infected with chikungunya virus, which mainly occurred if maternal viremia commenced at the time of delivery [105–107]. In women infected with chikungunya during pregnancy, viral antigens could be detected in placental tissue, including Hofbauer cells, along with a pro-inflammatory response, which provides emerging insights into the pathways underlying the vertical transmission [108, 109]. However, the small sample size of this study must be acknowledged, emphasizing the need to reproduce these findings in larger studies. Whilst often asymptomatic at birth, infected neonates developed symptoms such as fever, joint swelling and rashes within the first week after birth, in some cases, along with thrombocytopenia, petechiae and signs of liver damage [107]. Approx. 12% of previously chikungunya-infected neonates also show a significant developmental delay at the age of two 2 years [110], possibly caused by meningoencephalitis [111]. This highlights the potential long-term consequences of neonatal chikungunya infection and the urge to protect pregnant women from chikungunya infection, especially in the context of climate change and the spread of chikungunya.

#### Parasite-induced infections during pregnancy

Mosquitoes can also transmit parasites. One well-known example is malaria, an infection mainly transmitted by *Anopheles* mosquitoes. Five types of parasites can cause malaria, most commonly *Plasmodium falciparum* and *Plasmodium vivax*. At the onset of malaria, patients often show mild symptoms, such as fever and headache. However, malaria can also have a severe course, mirrored by jaundice, bleeding, and neurological symptoms, and even rapidly lead to death [64].

Every year, approximately 125 million women become pregnant in malaria-endemic regions [112]. Consequently, a significant number of pregnant women are at risk of malaria infection. Intriguingly, in high malaria transmission areas, plasmodia can be detected in blood samples from half of pregnant women [113]. Compared to non-pregnant women, pregnant women are more susceptible to malaria infection due to the preferential infection of the placenta by plasmodia [114, 115]. Here, plasmodia causes an inflammatory immune response, which is associated with impaired placental function and reduced fetal blood supply, resulting in fetal anaemia and low birth weight [116–118]. Malaria infections during pregnancy can even be fatal for both mother and child. Additionally, prenatal exposure to placental plasmodia increases the risk of malaria infection in early childhood, resulting in long-term consequences for the health of offspring [65].

Interestingly, nulliparous women are at the highest risk of malaria infection, while multiparous women develop resistance to plasmodia with each subsequent pregnancy, thereby gaining increasing protection from infection [119]. As described above, malaria outbreaks in Europe are still scarce, along with low herd immunity. Hence, if malaria became endemic in Europe, pregnant women would be highly predisposed to a severe course of malaria [119].

### Measures for the protection of pregnant women against vector-borne diseases

The screening and detection of viral infections are paramount in safeguarding the health of pregnant women and their offspring. For instance, in cases where infection with the Zika virus is confirmed, it is imperative to conduct regular ultrasound examinations to monitor fetal growth and development meticulously [120]. This approach facilitates the assessment of fetal well-being and allows for timely medical interventions if necessary. PCR laboratory diagnostics are essential in this context, especially if the mother shows signs of – often mild - infection. Detecting Zika virus infection necessitates timely diagnosis, ideally within seven days following the onset of clinical symptoms [120]. This requirement poses a significant challenge in clinical practice due to the variability and overlap of Zika’s symptoms with other viral infections. The complexity of accurately distinguishing Zika virus from other similar presentations can hinder effective and prompt diagnosis, emphasizing the need for improved awareness and diagnostic tools in healthcare settings. Serological tests that detect Zika virus-specific IgM, e.g. ELISA, are commonly employed for diagnostic purposes [120]. It is crucial to note that previous infection with a flavivirus can elevate the likelihood of cross-reactivity, which may result in false positive outcomes [121]. This potential for cross-reactivity necessitates careful interpretation of serological results in the context of the patient’s clinical history and exposure risks. The diagnostic challenges in arbovirus identification can be significant, mainly due to overlapping clinical features and the co-circulation of various arboviruses. This issue became particularly evident during the Zika epidemic from 2015 to 2017 in Latin America, where instances of misdiagnosis were not uncommon [122]. Such scenarios highlight the critical need for enhanced training and education for clinicians to improve diagnostic accuracy and patient care in the context of arboviral infections.

Vaccination is a crucial milestone in preventing infectious diseases and should play a significant role in combating the consequences of emerging mosquito-borne diseases. The rapid dissemination of Zika virus across continents over the last few years has accelerated vaccine development against Zika virus, with several phase 1 trials in progress [123–125]. Despite evident efforts to advance vaccine development, this process often faces delays due to the multiple phases required before individuals can effectively safeguard themselves against vector-borne infections. While data on the efficacy of these vaccines in pregnant individuals remains sparse, findings from mouse models indicate that vaccination before pregnancy effectively prevents vertical transmission [123].

Similar advancements have been made for West Nile virus. Although licensed veterinary vaccines exist, human vaccines have yet to progress beyond phase 1 or 2 trials. This lag can be attributed to vaccine design, the execution of efficacy studies, safety concerns, and high costs [126]. Once human vaccines become available, it is essential to include pregnant women in clinical trials from the outset due to the heightened risk of severe infection related to West Nile infection.

Currently, a vaccine for dengue in humans is approved [127]; however, it is not authorized for use during pregnancy due to insufficient data regarding its effect on fetal development and pregnancy maintenance. Inadvertent exposures to the vaccine during pregnancy have shown that adverse pregnancy outcomes occur in similar frequencies in vaccinated and non-vaccinated women, although the dataset is limited [128].

Lastly, a live vaccine against yellow fever is available; however, it is not advised for pregnant women. Those not immune to yellow fever virus should avoid travelling to regions where yellow fever is endemic. Unintentional exposure to the vaccine during early pregnancy did not increase the risk of adverse pregnancy outcomes [129].

### Outlook

A novel infectious disease that threatens human health occurs approximately every eight months [130]. Almost two-thirds of these diseases are caused by zoonoses. Pathogens causing malaria and dengue, but also West Nile fever, yellow fever and chikungunya infection, pose a significant health risk for pregnant women and their unborn children. Besides the viruses and parasites discussed here, additional pathogens are on the rise, which have not gained as much attention until now and are classified as tropical neglected diseases [53]. The potential threat of tropical neglected diseases must be investigated concerning highly vulnerable groups, e.g., pregnant women since the inevitable climatic changes promote their emergence.

Climatic changes are intimately linked to a wealth of factors determining our modern environment, such as increased travel and trade, deforestation, progressive urbanization due to population growth and others [131]. Slowing down or reversing these factors is one challenge. As researchers and physicians in the field of reproduction and infections, it is our responsibility to continuously express warnings that pregnant women and their unborn children are highly threatened by emerging infections resulting from climate change, modern environments and human behavioral shifts. These warnings can potentially increase the public’s awareness, which serves as an essential adaptation strategy [53, 132]. This is particularly relevant since pregnant women are vastly underrepresented in research endeavors investigating the health implications of climate change [14]. This lack of knowledge on the consequences of the infectious disease for the mother and fetus limits the development of guidelines. In this context, it is essential to fill research gaps on several mentioned aspects: for accurate estimation of risks of feto-maternal outcomes, large, controlled prospective cohort studies are needed with adequate confirmation of disease via RT-PCR in the mother and the newborn by, e.g. exanimating cord blood as well as the conformation via serological tests by IgM and IgG ELISA testing for seroconversion. Another aspect that should be considered in future research endeavors is the time point of infection.

Additionally, the molecular mechanisms must be understood to develop targeted therapeutic options. Studies are required to analyze the inflammatory responses on a systemic and local level (at the feto-maternal interface) and investigate the in-depth consequences for the placenta. Even if vertical transmission does not occur, the fetal response to cytokines and stress hormones is interesting.

Additionally, preventative measures must be taken. Vaccination is one of the most outstanding public health achievements of the last century, and vaccines administered during pregnancy protect mothers and children from infectious diseases. Safety and effectiveness of vaccination during pregnancy could be repeatedly shown, e.g. for influenza and COVID 19 vaccines during pregnancy [133, 134]. Yet, vaccine hesitancy is highly preponderant among pregnant women due to safety concerns [135]. This hesitance towards vaccines well-proven to be safe and highly recommended during pregnancy has caused a high degree of morbidity and even maternal and fetal deaths in the past. Thus, obstetricians must be aware of this threat and recommend vaccination to women during reproductive years and pregnancy.

Likewise, pregnant women are often omitted from vaccination trials against emerging pathogens. A very recent example is the initial exclusion of pregnant women from newly developed vaccination strategies against COVID-19 despite their higher risk for severe infections. Concerns about legal liability or ethical issues can justify such widespread omission of pregnant women and unborn children from vaccination trials. While these considerations are well-intended, consequences are fatal, mirrored by maternal and related neonatal morbidity in response to infections. If no vaccination is available or suitable during pregnancy, women—especially in European countries where the risk of vector-borne diseases is still underestimated—must be informed about other measures, such as the frequent use of repellent sprays.

In summary, climate-change-induced emerging infections pose a threat to our society. Since pregnant women and their unborn children are highly vulnerable to a severe course of infections, awareness must be raised to act towards the development of preventative measures rather than to react to increased morbidity and mortality caused by vector-borne infectious diseases.

## Data Availability

There is no additional data associated with this manuscript.
